# Ascorbic acid as serine protease inhibitor in lung cancer cell line and human serum albumin

**DOI:** 10.1371/journal.pone.0303706

**Published:** 2024-07-23

**Authors:** Bijon Kumar Sil, Mohd. Raeed Jamiruddin, Pijush Kumar Paul, Nattanit Aekwattanaphol, Titpawan Nakpheng, Md. Ahsanul Haq, Wilaiporn Buatong, Teerapol Srichana

**Affiliations:** 1 Drug Delivery System Excellence Center, Faculty of Pharmaceutical Sciences, Prince of Songkla University, Songkhla, Thailand; 2 School of Pharmacy, BRAC University, Dhaka, Bangladesh; 3 Immunobiology, Nutrition and Toxicology Lab, Nutrition Research Division, icddr,b, Dhaka, Bangladesh; Lahore University of Management Sciences, PAKISTAN

## Abstract

Serine proteases (SPs) are distributed among all living cells accounting for almost one-third of all proteases. Dysregulation of SPs during inflammation and/or infection can result in devastating consequences, such as skin and lung inflammation, neuroinflammation, arthritis, as well as metastasis of cancerous cells. Such activities are tightly regulated by various inhibitors known as serine protease inhibitors (SERPIN). The thermodynamic investigations previously revealed that L-ascorbic acid binds to trypsin more firmly than pepsin and the binding force of L-ascorbic acid is driven by hydrogen bonds and van der Waals forces. However, the physiochemical effects of such interaction on trypsin and/or pepsin have not yet been reported. Ascorbic acid, also known as vitamin C, is one of the essential nutrients and most common food supplements, fortificants, and preservatives. The aim of this study was to explore the inhibitory effects of ascorbic acid on serine proteases at various concentrations on the in-vitro digestion and/or hydrolysis of intercellular matrix of cell monolayer and human serum albumin (HSA). The inhibitory effects of ascorbic on trypsin are investigated by qualitative and quantitative analysis using SDS-PAGE imaging and NIH densitometric software. Upon the addition of ascorbic acid in both indicator systems, the detachment and/or dissociation of cell monolayer and the digestion of HSA were inhibited in the presence of EDTA-Trypsin. The inhibitory effect of ascorbic acid on the digestion of intercellular matrix and/or hydrolysis of HSA showed a dose-dependent trend until it reached the maximum extent of inhibition. At an equal concentration (2.5mg/mL) ascorbic acid and EDTA-Trypsin exhibited the most potent inhibitory effect on the in vitro digestion of protein either in the form of intercellular matrix in cell monolayer and/or HSA respectively. Overall, our results based on two indicator systems strongly indicate that ascorbic acid may function as a serine protease inhibitor (SERPIN) beyond other important functions.

## 1. Introduction

Serine proteases are being targeted for the design of enzyme inhibitors due to their involvement in the etiology of several diseases. Within the class of serine proteases, human leukocyte elastase (HLE) is one of the most destructive groups of enzymes in the body [1, 2]. Neutrophil elastase (NE) also plays a significant role in increasing vascular permeability and leukocyte transmigration [3]. Additionally, NE has been reported to have the capacity to cleave E-cadherin and interfere with its cell-cell adhesion function which can lead to disease resulting in tissue destruction and lung inflammation [4].

Trypsin, secreted in its inactive form as trypsinogen in the pancreas, has been implicated in the regulation of various bodily functions including digestion and immune response [5]. It has been identified as the major member of the serine protease group [6]. Markus et. al. first presented evidence for the hydrolysis of serum albumin by trypsin. Digestion of the human serum albumin at pH 8.8 results in the formation of a trypsin-resistant fragment capable of binding to bilirubin and diazepam [7]. Additionally, hydrolysis of albumin with trypsin results in three to four fragments of albumin [8–10]. Similarly, albumin has also been implicated with serine protease activity towards 4-methylumbelliferyl-guanidinobenzoate (MUGB) and diisopropyl fluorophosphate (DFP) [11].

Serine protease inhibitors (SERPINs) are a class of proteins involved in the regulation of serine proteases [12, 13]. Due to their ubiquity in regulating various bodily functions, various diseases have been purported to be the dysfunction of serpins [14]. There have been other non-serpin molecules that have been observed to regulate serine proteases. Ascorbic acid, commonly known as vitamin C, is a water-soluble micronutrient essential for the proper functioning of the body. It can be found in many foods, particularly fruits and vegetables such as citrus fruit, broccoli, and spinach. It is required for the formation and maintenance of connective tissues and serves as a potent antioxidant that protects the body from harmful free radicals [15]. Since it is water-soluble, excess ascorbic acid is easily excreted in urine and rarely accumulates to toxic levels. Such property makes the utilization of ascorbic acid advantageous over medications that may cause adverse symptoms at high doses. Beyond being required for the aforementioned essential metabolic activities, ascorbic acid binds to trypsin more firmly than pepsin, at equimolar concentration, while the binding force of L-ascorbic acid is driven by hydrogen bonds and van der Waals forces [16]. Moreover, few studies have confirmed the inhibitory effects of ascorbic acid in pancreatic α-amylase, a digestive enzyme that plays a major role in breaking down starch into glucose, via noncompetitive antagonism [15]. The SDS-PAGE analysis was applied to monitor the hydrolysis process of protein as a substrate because the polyacrylamide gels were an effective tool for the detection of hydrolyzed protein bands [17–19]. The densitometric analysis of SDS-PAGE using NIH software was also established as a good indicator to define the color intensity of desired bands [16].

In this study, we explore the role of vitamin C in regulating the enzymatic activity of trypsin, a serine protease using two indicator systems such as cell monolayer and human serum albumin (HSA) using two serine proteases (EDTA-trypsin and elastase). EDTA-trypsin is a widely used enzyme in cell culture laboratory for the dissociation of cells from the surface of cultured vessels while elastase is one potent enzyme secreted by neutrophil [3]. The SDS-PAGE analysis was used for the identification of HSA hydrolyzed proteins using the standard protocol provided by the manufacturer. Both qualitative and quantitative analysis were conducted by imaging system and densitometric analysis using NIH software.

## 2. Experimental procedures

### 2.1. Chemical and reagents

EDTA-Trypsin [EDTA (0.02%), Trypsin (0.25%)] solution was obtained from Gibco^TM^ (Cat. No. 25200056) (Gibco, USA), which will be referred to as trypsin hereafter, and used as catalytic enzyme for the dissociation of cell monolayer and/or digestion of human serum albumin (HSA) (Cat. No. A9511) (Merck, USA) obtained in powder form from Merck. Elastase was obtained from Sigma-Aldrich (Cat. No. E0127) (Sigma-Aldrich, USA). L-ascorbic acid was obtained from ChemSupply (Cat. No. AA022) (CSAScientific, Australia) in a crystalline form and used as SERPIN. The A549 (ATCC CCL-185) (ATCC, USA) human lung carcinoma cell line was obtained from ATCC and used as one of the indicator systems to study the SERPIN activity of L-ascorbic acid. The SDS-PAGE pre-cast gels (Cat. No. M00667), Tris-MOPS-SDS as running buffer (Cat. No. M00138) and loading buffers (Cat. No M00676) were obtained from GenScript USA Inc. Protein marker for SDS-PAGE were obtained from Energenesis Biomedical Co. Ltd (Cat. No. VC03-250/VC03-500). Staining buffer was prepared using sodium dodecyl sulfate (SDS) (Thermo Fisher Scientific, USA), β-mercaptoethanol (Merck, USA), and bromophenol blue (Thermo Fisher Scientific, USA). The SDS-PAGE system was carried out using the GenScript SurePAGE^TM^ gels protocol. Destaining buffer was prepared using AR grade in both methanol (Merck, USA) and acetic acid (Merck, USA). Imagequant LAS 500 (Cytiva, USA) was used for imaging of gel and NIH densiometric software (ImageJ, NIH, USA) was used for band analysis of the gel and color intensity of cell monolayer following staining with crystal violet (Cat. No. AC405830250) (Thermo Fisher Scientific, USA).

### 2.2. Preparation of plates and screening of protease inhibitor in human lung carcinoma A549

The A549 (ATCC CCL-185) human lung carcinoma cell line (10^5^ cells/well) was cultivated in a 6-well plate using standard technique until fully confluent. Once ready the culture medium was removed, and the cells were washed twice with PBS (typically 100–200μl). Following the wash, cells were incubated at 37°C for 2 minutes with PBS as experiment control, trypsin and elastase only, and ascorbic acid along with trypsin and elastase in their designated wells. In every experiment, cell controls were kept where only 200μl of PBS was added. The plates were observed at 5-minute intervals to observe changes in cell morphology. Once the cells of the trypsin-treated wells were lifted off from the surface, fluid from all wells was discarded, washed two times with PBS (typically 100–200μl), and stained with 1% crystal violet (in alcohol) for three minutes. Then the stains were removed, and the wells were washed with PBS to remove excess dye. Finally, cell monolayers of treated and control wells were checked with the naked eyes and cell morphologies were observed and recorded with an inverted microscope. The color density was calculated using densitometric software. Identical steps were carried out to observe the effects of elastase and L-ascorbic acid obtained as a health supplement.

### 2.3. Progression of dissociation and/or inhibition of cell monolayer by trypsin and ascorbic acid

To observe for the dissociation the plates were prepared and incubated as previously mentioned. Both the dissociation of the A549 by trypsin and its inhibition by ascorbic acid were observed using an inverted microscope (Olympus CKX41, Japan). The changes were recorded using EOS-700D (Canon, Japan) at minute intervals until the cells in the trypsin-treated wells dissociated from the plate surface. The morphology of the cell monolayer without treatment (L-ascorbic acid and EDTA-Trypsin (ET)) was also recorded and the color density was calculated.

Titration of ET was carried out in confluent A549 cells (10^6^/well) using varying concentrations of ET ranging from 5.0 mg/mL to 0.156 mg/mL per well using the aforementioned protocol. Following 5–7 minutes of incubation at 37°C cells were treated with 2% crystal violet for 2–3 minutes. Two separate rows of cells in 6 replications were kept as cell control and ET control (2.5mg/mL). Following staining cells were washed 3 times with PBS and the result was recorded and the densities of stained using NIH densitometric software.

### 2.4. Determination of minimum inhibitory concentration of ascorbic acid and its serine protease inhibitor activity

Ascorbic acid was dissolved in deionized water to obtain a stock solution containing 100mg (567.8mM). The stock solution was then diluted to 2mg (113.6μM), 1mg (56.8μM), 0.5mg (28.4μM), 0.25mg (14.2μM), and 0.125mg (7.1μM) in PBS. Each concentration of ascorbic acid was added to 0.25 mg (0.107μM) concentration of trypsin into the wells with monolayers of A549 which were prepared as previously described. The endpoint of trypsin inhibition by ascorbic acid was calculated in terms of mg/100μl.

In case of serine protease inhibitory activity of L-ascorbic acid in HSA-Trypsin hydrolysis 100μl of L-ascorbic acid (2.5 mg/mL) was mixed with an equal volume and concentration of ET (2.5mg/mL) and added into 100μl of HSA (2.5mg/mL) of HSA and then treated at two temperatures (37°C & 40°C) in water bath for 10 to 20 minutes. HSA (2.5mg/mL) without L-ascorbic acid and ET was also treated under similar conditions as a control. Similarly in the case of elastase, 1.25mg/mL of ascorbic acid was treated with an equal concentration of elastase. Finally, the solutions were subjected to SDS-PAGE for semi-quantitative analysis using the aforementioned protocol.

### 2.5. Determination of optimum concentration of HSA and its hydrolysis by serine proteases (trypsin and elastase)

HSA was dissolved in double distilled water to obtain a stock solution of 150 μM (10 mg/ml). The stock solution was then diluted in two-folds of serial dilutions in PBS to obtain, 75 μM (5 mg), 37.5 μM (2.5 mg), 18.75 μM (1.25 mg), and 9.38 μM (0.625 mg). 100 μl of each dilution was mixed with 100 μl ET and elastase (1.25% w/v) and incubated in the water bath at 40°C for 10 minutes. After the incubation, 22.5 μl of sample was added to 7.5 μl of loading buffer solution. The hydrolysis dynamic of HSA by trypsin was studied in 12% precast Tris-glycine gel. 10μl of sample from each dilution was subjected to SDS-PAGE for semi-quantitative analysis.

### 2.6. Determination of minimum inhibitory concentration of ascorbic acid and its serine protease inhibitor activity in A549 cells and human serum albumin (HSA)

Ascorbic acid was dissolved in deionized water to obtain a stock solution containing 100mg (567.8 mM). The stock solution was then diluted to 5.0 mg/mL (28.38μM), 2.5mg/mL(14.19μM), 1.25mg/mL (7.097μM), 0.625mg/mL (3.54μM), 0.312mg (1.7μM), and 0.156 mg (0.885μM) in PBS. Each concentration of ascorbic acid was added to 0.625mg/mL (μM) concentration of trypsin into the wells with monolayers of A549 (10^6^ cells/well) which were prepared as previously described. The endpoint of elastase inhibition by ascorbic acid was calculated in terms of mg/100μl.

### 2.7. SDS-PAGE and semi-quantitative analysis

14 μl of sample from the treatment was added to the Eppendorf tube containing 6μl of loading buffer solution which was totaled to 30 μl by addition of distilled water. The final resulting product was incubated at 70°C in the water bath for 10 minutes. 5μl of protein ladder and 10μl of each sample were loaded into their designated lane in pre-cast gel and were subjected to a runtime of 60 minutes at 120V. After the completion of the run, the gels were stained with Coomassie brilliant blue for 15 minutes. Following overnight destaining, Gels were digitized using ImageQuant^TM^ LAS500 (GE, USA) which was later subjected to semi-quantitative analysis using densitometric software (NIH).

### 2.8. Determination of optimum temperature for serine protease inhibition of HSA-trypsin hydrolysis at four-time point of treatments

100μl of trypsin (2.5 mg/mL) was added into an Eppendorf tube containing an equal volume of HSA (2.5 mg/mL) and then treated to 37°C in the water bath at four different time intervals of 5, 10, 15, and 20 minutes. Similarly, other sets of four EP tubes were prepared each set containing 100μl of trypsin (2.5 mg/mL) and an equal volume of L-ascorbic acid (2.5mg/mL) and then 100μl of HSA (2.5 mg/mL) was added. The total volume of each set of Eppendorf tubes becomes 300μl and incubated at 37°C or 40°C in the water bath at four different time intervals of 5, 10, 15, and 20 minutes. In both cases, the treatment was stopped by inserting the Eppendorf in ice. Finally, 14 μl of sample from each tube was subjected to SDS-PAGE as mentioned previously. HSA control was kept (without HSA and L-ascorbic acid) in each run and subjected to a similar treatment.

### 2.9. Determination of serine protease inhibition effects of ascorbic acid by HSA-elastase hydrolysis at 40°C

50μl of elastase (0.625mg/mL) was added into the Eppendorf tube containing an equal volume (50 μl) of HSA (2.5 mg/mL) and then treated to 40°C in the water bath at 10 min. Similarly, other sets of four Eppendorf tubes were prepared each set containing 50μl of elastase (0.625mg/mL) and an equal volume of L-ascorbic acid (2.5mg/mL) and then 50μl of HSA (2.5 mg/mL) was added. The total volume of each set of Eppendorf tubes becomes 150μl and is incubated at 40°C in the water bath for 10 minutes. In both cases, the treatment was stopped by inserting the Eppendorf in ice. Finally, 14 μl of sample from each tube was subjected to SDS-PAGE as mentioned previously. Both HSA and elastase controls were kept in each run and subjected to similar treatment.

### 2.10. Observation of cell morphologies, calculation of color densities, and quantification of gel image

In all experiments, the cell morphologies were observed using an Olympus inverted microscope (Olympus, Japan). The color densities in all experiments were obtained and calculated using the densitometric function of NIH ImageJ software (NIH, USA). Similarly, images of gels were read by Image quant LAS 500 and recorded for quantification. The density of bands was obtained through gel image and calculated through NIH ImageJ software (NIH, USA) and semi-quantitative analysis was carried out.

### 2.11. Statistical analysis

The linear association between exposure and outcomes was assessed using a simple linear regression model. Additionally, the concordance correlation coefficient (CCC) between the two assays on different days was examined. For data analysis and figure preparation, we utilized GraphPad Prism 8.3.2, STATA (version 15), and Python-3.11 within Jupyter Notebook.

### 2.12. Ethical approval and/or consideration

The study does not include the usage of any human or animal samples (REF: PSU 68108.7/980). Furthermore, all the experiments were carried out using laboratory standard protocols and equipment (REF: PSU 68108.7/981).

## 3. Results

### 3.1. Anti-serine protease activity of ascorbic acid against ET and elastase

**Fig 1:**
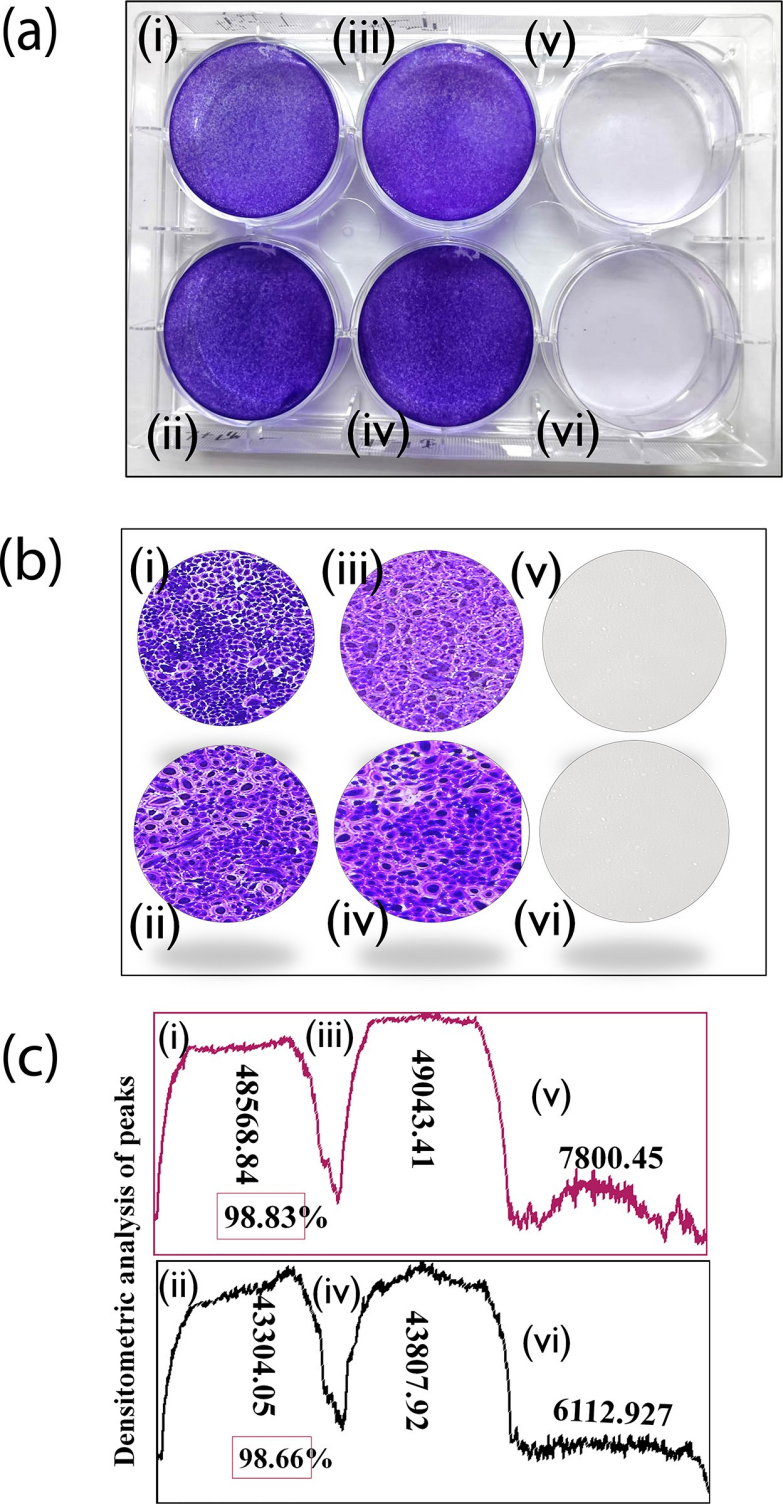
Screening of inhibitory effects of L-ascorbic acid against serine proteases (trypsin and elastase). (a) Following crystal violet staining both L-ascorbic acid treated and control cell monolayers show a good and uniform stained cell against enzymes (trypsin and elastase) while wells treated with enzymes completely lift off after 5 minutes of treatment. (b) Cell morphologies under microscopic examination showed good infirmity in both control and L-ascorbic acid treated cells (c) densitometric analysis of cell monolayer and showed >98% enzymes are inhibited by L-ascorbic acid compared to control groups (cell and enzymes). [(i) A549 cells treated with ET and L-ascorbic acid; (ii) A549 cells treated with elastase and L-ascorbic acid; (iii) & (iv) Cell control without any treatment (A549); (v) A549 cells treated with ET; (iv) A549 cells treated with elastase].

On plating the A549 cells form a monolayer on the bottom surface of the plate which is indicative of the uniformity of the stain. The addition of the ET or elastase results in the cells being detached from the surface of the well which can be observed with the lack of stained cells in the well (Fig 1(A) and 1(C)). However, with the addition of ascorbic acid, it is observable that the densities of stained cells are similar to that of cell control (Fig 1(B)). This may be indicative of the anti-serine protease activity of the ascorbic acid upon ET and elastase.

**Fig 2:**
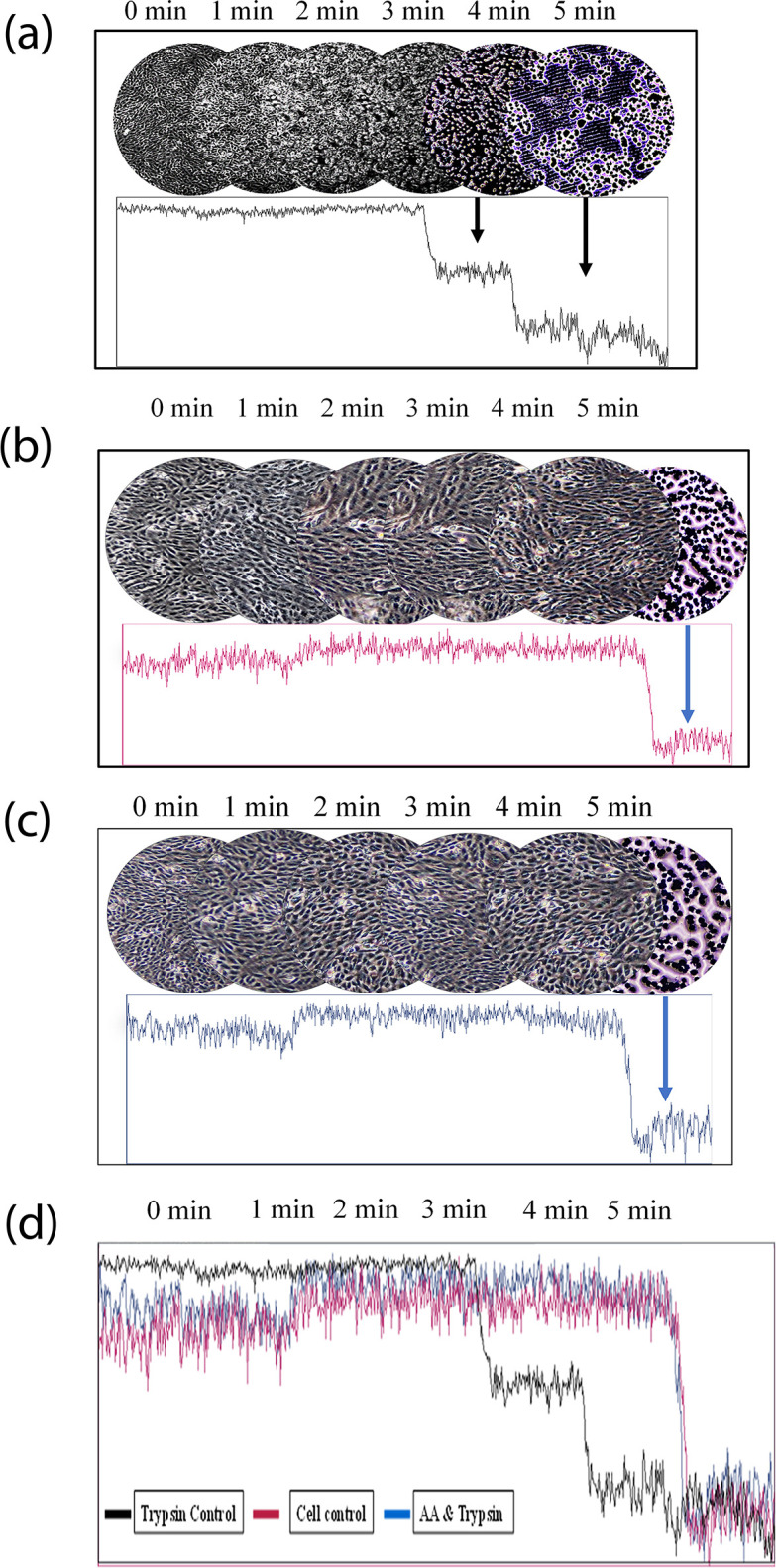
Studies of the progression of dissociation of cell monolayer by ET and their inhibition by L-ascorbic acid. (a) cell monolayer treated with ET and monitored under the microscope at every 1-minute interval. Cells presented higher refractility till 3 minutes. At 4-minute mark, the cell lines started showing lower refractility with complete detachment at 5 minutes. The uniformity of cells showed good integrity measured by densitometric analysis. (b) Cells in the cell control group remained unaffected and showed uniform intensities. (c) Cells in the L-ascorbic acid-treated cell monolayer also showed similar types of cell morphologies and color intensities to that of the control cell monolayer. (d) comparative analysis of color densities of three groups of cell monolayer.

Microscopic observation of time-dependent dissociation progression of cell monolayer realized that the cellular integrity was intact till 3 minutes, based on the refractility of cells in the case of ET-treated cells monolayer. However, low refractility was observed at 4 minutes with a complete lack thereof at 5 minutes at room temperature (Fig 2(A)). The refractility and uniformity of the control cell monolayer as well as the cell monolayer that has been treated with ET and ascorbic acid, are similar as evident from the densitometer (Fig 2(B) and 2(C)). The merging of densitometric analysis of three treatments showed close cellular integrities between cell control and L-ascorbic acid and ET treated wells while in ET control cell monolayer lost integrities after 4 minutes of treatment (Fig 2(D)).

### 3.2. Determination of concentration of ET or elastase with L-ascorbic acid for 50% enzymatic inhibition in a cell line (A549)

We determined the 50% dissociation of ET for A549 cells to be between 0.625 mg/mL and 0.312 mg/mL. However, at 2.5 mg/mL, we observed that the cells presented more than 85% of dissociation, due to which we determined the concentration for complete dissociation to be at 2.5 mg/mL instead of 1.25 mg/mL (S1(A) and S1(B) Fig). In the case of elastase, 50% dissociation was determined at a concentration between 0.625 mg/mL and 0.312 mg/mL. Henceforth, we determined the concentration of elastase for the complete dissociation of the cells to be 1.25 mg/mL (S2(B) Fig).

**Fig 3:**
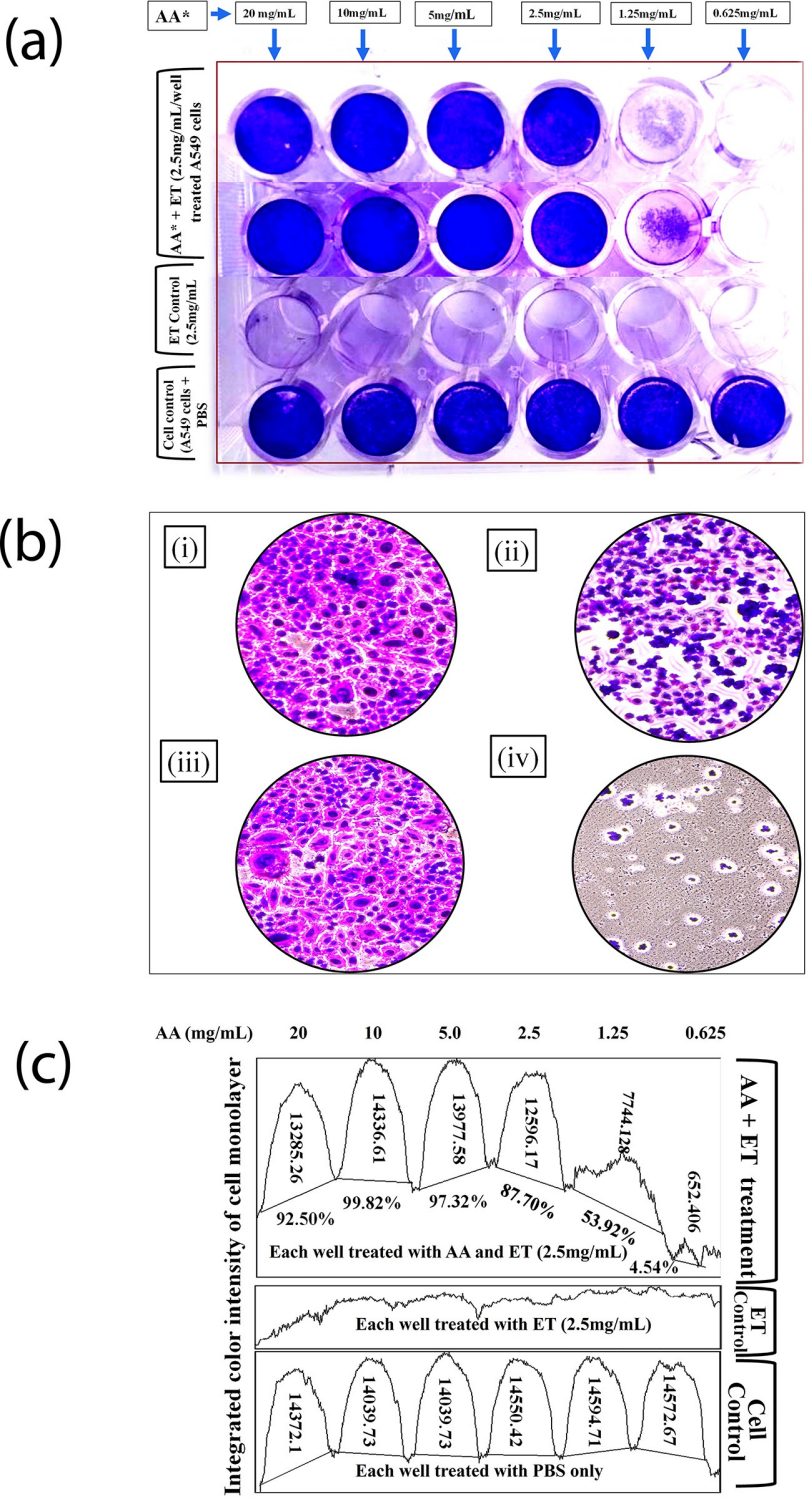
Determination of 50% inhibition of ET activities by L-ascorbic acid. (a) cell monolayers following staining with crystal violet. (b) Morphological characteristics of cell monolayers following crystal violet staining under the microscope. (c) densitometric analysis of color intensity and % of inhibition compared to ET and cell controls. A 53.92% inhibition was observed at 1.25mg/mL of L-ascorbic acid in the presence of 2.5mg/mL of ET. [(i) & (ii) A549 cells treated with ET and L-ascorbic acid; (iii) Cell control (A549); (iv) A549 cells treated with ET].

The fifty percent (50%) inhibition of the dissociation of cell monolayer by L-ascorbic acid in the presence of ET was achieved at the concentration of 1.25mg (Fig 3(A)) while in case of elastase, it was achieved at the concentration between of 0.625mg/mL and 0.312mg/mL (S3(A) and S3(B) Fig). The cell morphologies were also observed and found to be uniformly distributed with intact cell boundaries and comparable with the cell control monolayer (Fig 3(B); S3(A) Fig). The densitometric analysis of color taken by cells was measured using NIH densitometric software and showed 53.92% of enzymatic inhibition was achieved at the concentration of 1.25mg of L-ascorbic acid (Fig 3(C)) against 2.5 mg/mL of ET. In the case of elastase, 45.37% of inhibition was achieved between the concentration of 0.625 mg/mL and 0.312 mg/mL. Therefore, for complete inhibition, we were able to determine 1.25 mg/mL concentration of L-ascorbic acid against 1.25 mg/mL of elastase (S3(B) Fig).

**Fig 4:**
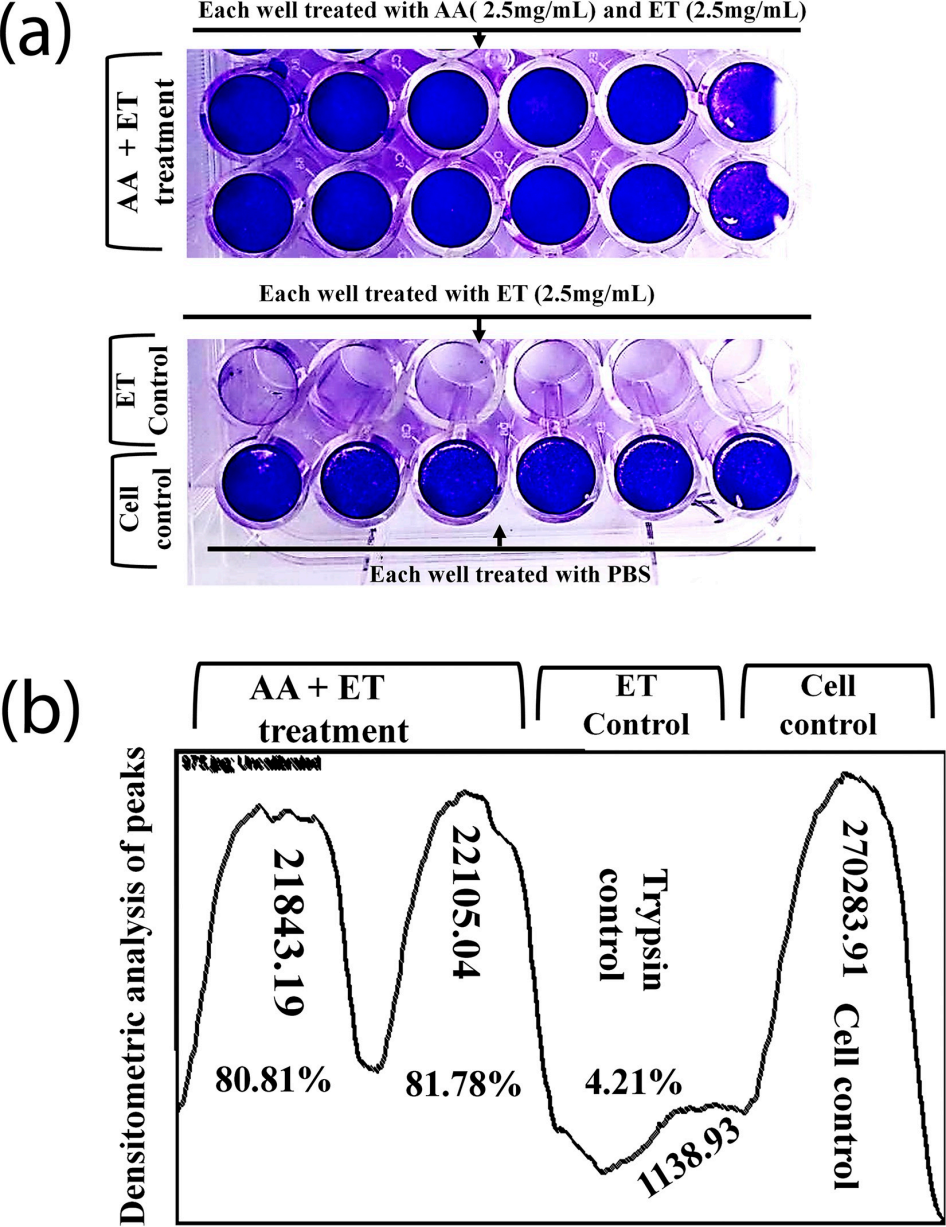
Validation of L-ascorbic acid SERPIN activity in cell monolayer with an equal concentration of ascorbic acid (2.5mg/mL) and ET (2.5mg/mL). (a) 12 replicates (6 in each row) of AA+ET treated samples showed >80% inhibitory effects compared to 6 ET and 6 cell monolayer controls. (b) Color intensities of cell monolayer following staining with crystal violet and measured by NIH densitometric software [AA (L-ascorbic acid); ET (EDTA+ Trypsin)].

Furthermore, the validity of inhibitory effects of both L-ascorbic acid and ET was 2.5mg/mL while that for both the L-ascorbic acid and elastase was 1.25 mg/mL, as determined previously, using 12 replicates (Fig 3 and S3 Fig). Following staining with crystal violet, the visual examination showed uniformity of inhibition (Fig 4(A) and S3(A) Fig) which is further confirmed by the densitometric analysis peak of color densities of cell monolayers in comparison with the cell and ET or elastase control monolayer (Fig 4(B) and S3(B) Fig). The average inhibition at an equal concentration of L-ascorbic acid (2.5mg/mL) and ET (2.5mg/mL) was found to be >80% while in the case of L-ascorbic acid (1.25mg/mL) and elastase (1.25mg/mL) was found to be >99.89%.

### 3.3. Temperature dependent activity of ET, human serum albumin and ascorbic acid

**Fig 5:**
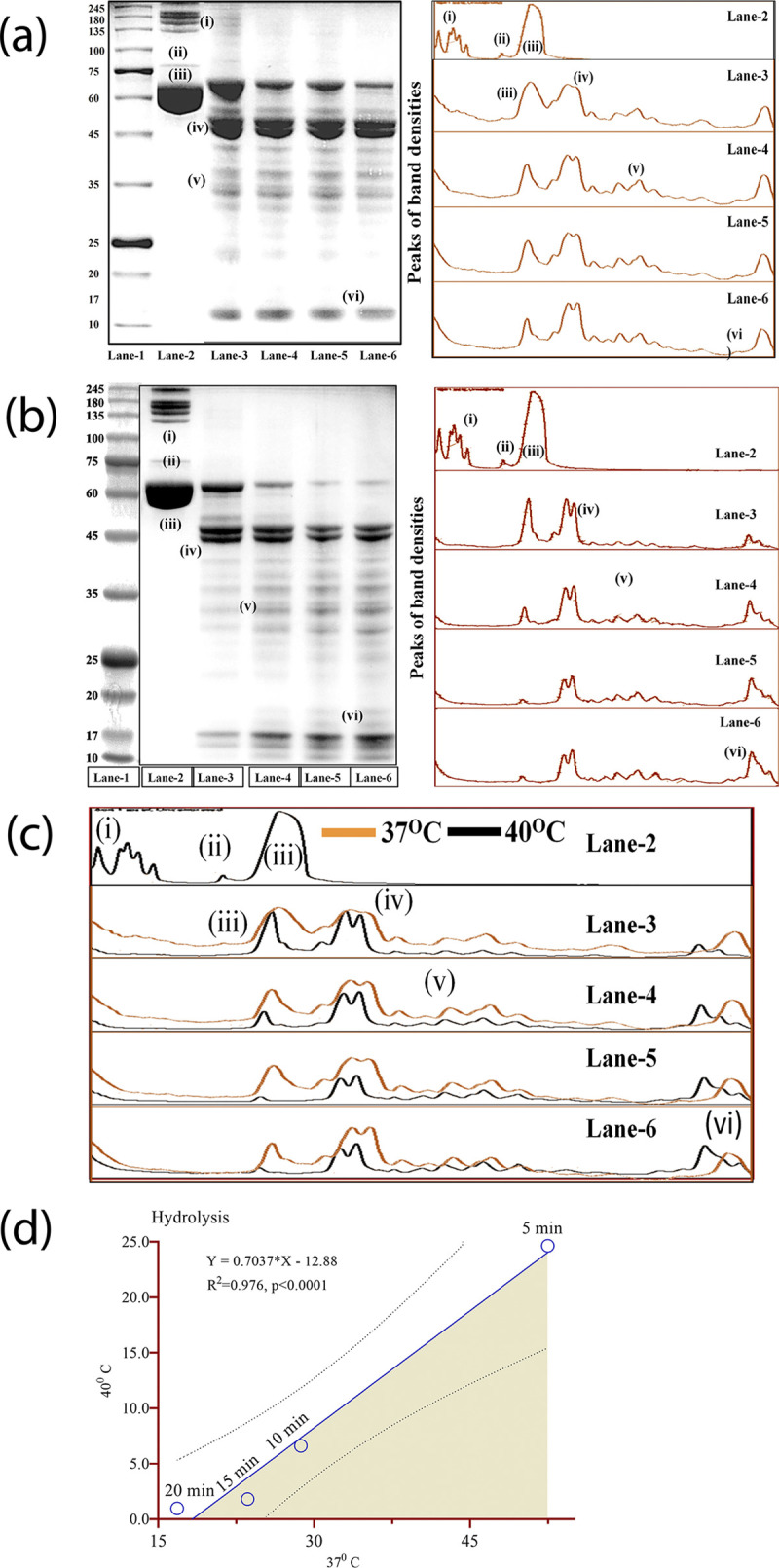
Analysis of images of hydrolyzed human serum albumin by ET. (a) Both qualitative (image) and quantitative (densitometric) analysis of protein bands following treatment at 37°C using four different time points (5, 10, 15, and, 20 mins) shows similar patterns of digestion and are closely related with the time of treatment. Four major groups of bands are observed [(iii)-(vi)] which formed following digestion of the main HSA protein (iii) and other two proteins [(i) and (ii)]. (b) Similar patterns of protein digestion are observed following 40°C of treatment but show better digestion compared to 37°C and almost 99% of main HSA is digested after 20 minutes of treatment. (c) Merging of peaks of bands observed at two temperatures (d) Graphical presentation of digestion of HSA bands at two different temperatures (37°C and 40°C). [Lane-1 = protein markers (245kDa-15kDa); Lane-2 = HSA protein; Lane-3 = HSA treated with ET at 5 minutes; Lane-4 = HSA treated with ET at 10 minutes; Lane-5 = HSA treated with ET at 15 minutes; Lane-6 = HSA treated with ET at 20 minutes] [(i) = High molecular proteins (245kDa-100kDa); (ii) = Upper HSA band; (iii) = Main HSA band; (iv-vi) = Hydrolysed HSA bonds)].

Trypsin activity was observed to be influenced by temperature and the period of treatments. It can be observed that trypsin at 40°C presented a higher percentage of HSA hydrolyzed at all time points of treatment when compared with the 37°C and continued such trends up to 20 minutes (Fig 5). Both qualitative and quantitative analyses showed similar trends of hydrolysis and are supported by previous findings [20]. In the HSA control protein (Fig 5(A) and 5(B): Lane-2) there are two sets of bands that are formed namely the higher molecular weight protein (HMWP) and HSA (Fig 5(A)). It can be observed that within the first 5 minutes of exposure to trypsin (Fig 5(A) and 5(B): Lane-3) there is a formation of 4 sets of bands which keep increasing with the increase of the period of treatment (5–20 minutes) it can be observed that the second band (Fig 5(A): iii), which represents the main HSA protein has reduced in concentration while the following higher band has increased. The highest increase is the small molecular weight protein (Fig 5(A) & 5(B): iii) is indicative of complete digestion of the HSA protein. This experiment confirmed that 40°C is the optimum temperature while the highest level of hydrolysis was observed after 15 minutes of treatment. Another important observation was that high molecular weight protein bands (Fig 5: (i)) (245–100 kDa) were completely digested at all time points of treatment (Fig 5(A) and 5(B)) and so digested protein bands (Fig 5(A) and 5(B): iv-vi) could be a result of digestion of either HMWP or HSA or both. Merging of peaks of densities of protein bands using both temperatures showed clear demarcation which was further confirmed in graphical presentation (Fig 5(C) and 5(D)).

### 3.4. HSA-Trypsin and HSA-elastase hydrolysis and its percentage inhibition by ascorbic acid

**Fig 6:**
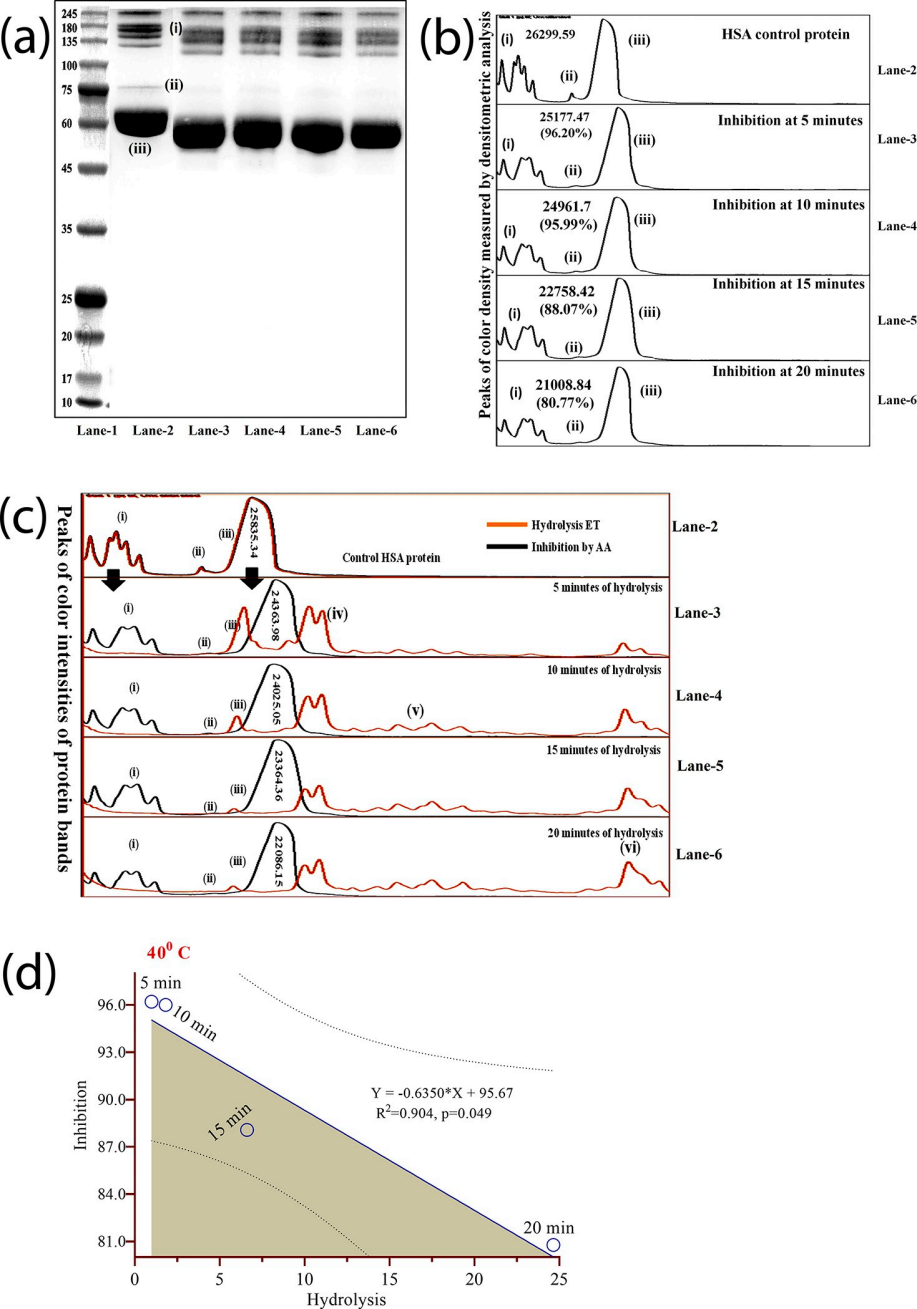
Inhibition of ET hydrolysis of HSA by L-ascorbic acid. (a) Gel image showing HSA and the inhibition of its hydrolysis by L-ascorbic acid at four different time points. (b) The densitometric analysis of peaks of protein bands. (c) Comparative densitometric analysis between hydrolysis of HSA and its inhibition by L-ascorbic acid. (d) Graphical representation of hydrolysis and inhibition (R^2^ = 0.904; *p-value* = 0.049). [Lane-1 = protein markers (245kDa-15kDa); Lane-2 = HSA protein; Lane-3 = HSA treated with ET at 5 minutes; Lane-4 = HSA treated with ET at 10 minutes; Lane-5 = HSA treated with ET at 15 minutes; Lane-6 = HSA treated with ET at 20 minutes] [(i) = High molecular proteins (245kDa-100kDa); (ii) = Upper HSA band; (iii) = Main HSA band; (iv-vi) = Hydrolysed HSA bonds)].

The SERPIN activity of ascorbic acid was conducted at four different points of treatment (5–20 minutes), at 40°C using the aforementioned protocol. Following qualitative and quantitative analysis it was observed that ascorbic acid inhibited both groups of proteins (Fig 6(A)–6(C): i & iii) from the hydrolysis of trypsin (Fig 6(A) and 6(B)), although a certain level of hydrolysis was among high molecular weight protein group (Fig 6(B) and 6(C): Lane 2–6: (i)), in contrast, a good degree of inhibitions was observed in the HSA band (96.2% to 80.77%) (Fig 6(A)–6(C): Lane 2–6: (iii)), although the band shifted a bit to the lower molecular weight, it could be due to some digested proteins from group higher molecular weight proteins (Fig 6(A)–6(C): i & ii). These changes were only observed within the first 10 minutes, after which there was a stabilization of the band formation.

**Fig 7:**
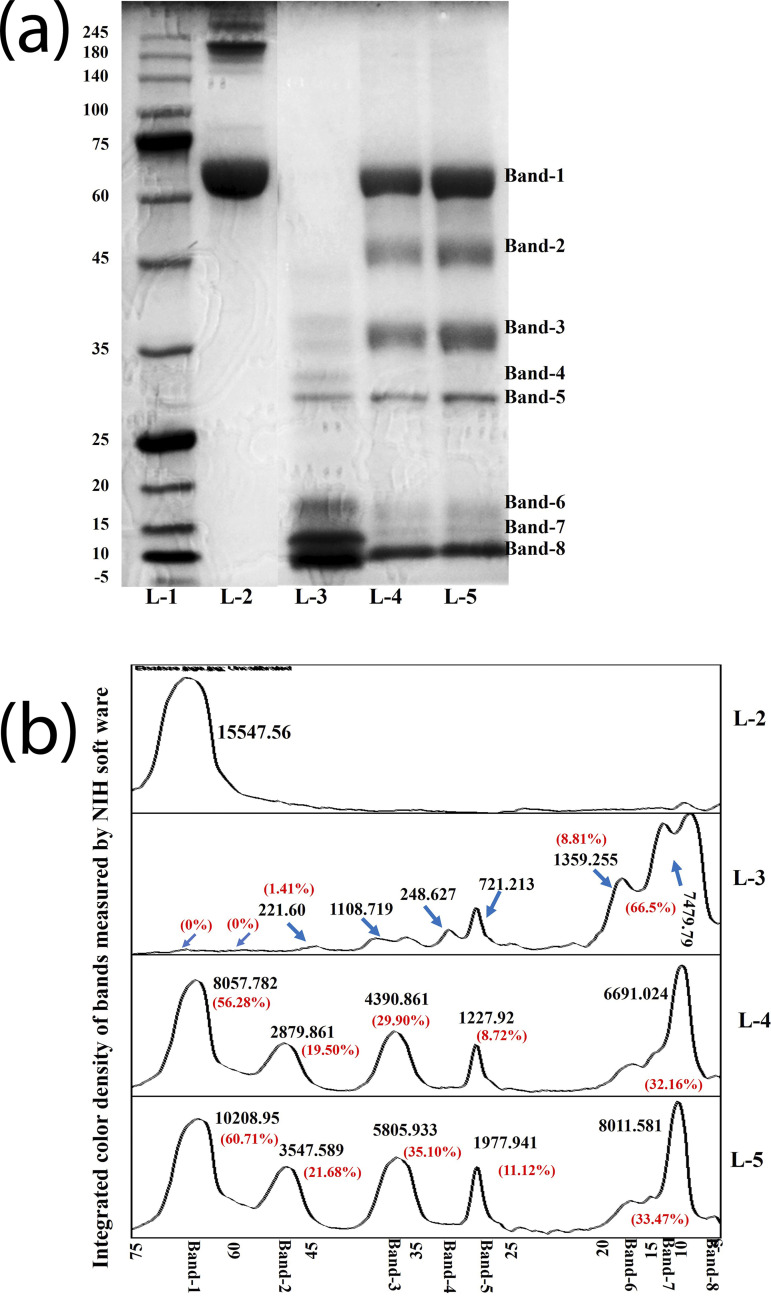
Inhibition of elastase hydrolysis of HSA by L-ascorbic acid. (a) Gel image showing HSA and the inhibition of its hydrolysis by L-ascorbic acid at 10 minutes at 40°C. (b) The densitometric analysis of peaks of protein bands. [L-1 = protein markers (245 to 5 kDa); L-2 = HSA protein; L-3 = HSA treated with elastase at 10 minutes; L-4 & L-5 = HSA treated with elastase and L-ascorbic acid at 10 minutes] [Band-1 = HSA control; Band-2 to Band 8 = digested peptides].

Since the SERPIN activity of ascorbic acid against ET was observed to be effective within the first 10 minutes at 40°C, we observed the SERPIN activity of L-ascorbic acid against elastase at that time point and at that temperature only. L-ascorbic acid was able to inhibit hydrolysis of HSA in its presence by 58.50% (Fig 7: L-4 & L-5: Band-1) in contrast to enzyme control which was able to carry out 100% digestion of HSA protein (Fig 7: L-3: Band-1). The partial inhibition can observed with the formation of digested peptides (Fig 7: L-4 & L-5: Band-2 to Band-8).

### 3.5. Comparative analysis of inhibition of HSA-Trypsin-EDTA in the presence of ascorbic acid at 37°C and 40°C temperatures

**Fig 8:**
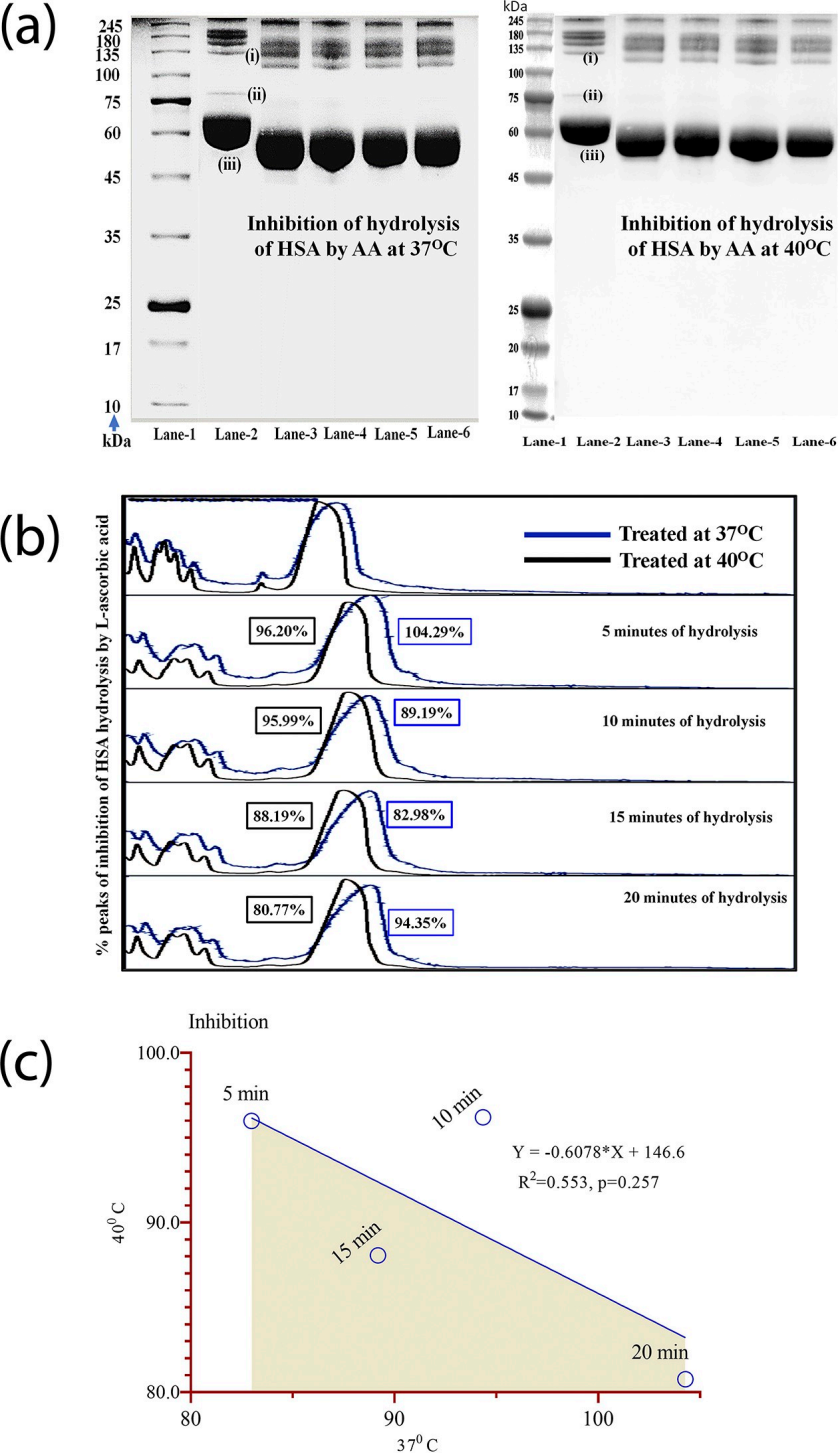
Comparative analysis of inhibition of HSA digestion by ET in the presence of L-ascorbic acid at 37°C and 40°C. (a) Gel image of inhibition of HSA hydrolysis at four time points (5 minutes, 10 minutes, 15 minutes, and 20 minutes) at two temperatures. (b) Densitometric analysis of merged peaks of HSA hydrolysis inhibition at two temperatures. (c) Graphical presentation of degree of inhibition at four time points of treatment which shows better correlation at 40°C compared to 37°C. [Lane-1 = protein markers (245kDa-15kDa); Lane-2 = HSA protein; Lane-3 = HSA treated with ET at 5 minutes; Lane-4 = HSA treated with ET at 10 minutes; Lane-5 = HSA treated with ET at 15 minutes; Lane-6 = HSA treated with ET at 20 minutes].

A qualitative and quantitative comparative analysis of inhibition of HSA by ET in the presence of ascorbic acid was carried out using the aforementioned protocol at four different time points of treatments using two temperatures (37°C and 40°C) (Fig 8). Upon comparison of the densitometric data point it was observed, that there was good inhibition varying from 80.77% to 105%. However, there was almost no correlation between the inhibition at 37°C and 40°C.

## 4. Discussion

This study is targeted to explore the possible anti-serine protease activity of ascorbic acid. The approach is based on the observation of inhibitory effects of L-ascorbic acid on trypsin and elastase using two biological indicator systems such as cell monolayer and human serum albumin. The qualitative and quantitative analysis of anti-trypsin and anti-elastase effects of L-ascorbic acid clearly showed that L-ascorbic acid can inhibit the hydrolysis of intercellular bridges in cell monolayer and HSA caused by ET as well as elastase. The inhibition of L-ascorbic acid could be driven due to the tight bonding of the hydroxyl group of L-ascorbic acid from positions 2 and/or 3 with histidine-57 of the enzyme. Histidine is in position to act as a base, a proton acceptor, and remove the proton from the OH group of serine. With this change, the serine is much more reactive, and can easily form a new bond with the carbon atom in the peptide bond of HSA and initiate the hydrolysis process. HSA is also rich in lysine and arginine amino acids which appropriately fit the protein in the trypsin catalytic pocket where negatively charged aspartic acid forms a bond with positive charged amino acids (arginine and lysine) of HSA and completes the specific spatial arrangement in enzyme pocket [21]. This spatial arrangement is found in hundreds of enzymes and is often referred to as "the catalytic triad" [22]. Our findings indicated the underlying mechanism that could be owing to the hydroxyl groups present in the ascorbic acid molecules and may be crucial in binding to trypsin and elastase. Similar mechanisms are observed in other enzymes such as amylase leading to inhibitory activity [23, 24].

One of the previously published reports has also claimed that ascorbic acid formed a stronger bond with trypsin than pepsin and proved that the main driving force of such binding comes from the hydrogen bond and van der Waals force [19]. Additionally, our inhibition test shows that ascorbate inhibits the trypsin while ascorbic acid has half maximal inhibitory concentration (IC_50_) in a cell culture monolayer of about 1.25mg/mL (7.09μM)

Furthermore, we observed that when the concentration of ascorbic acid decreases to the level where optimum saturation of hydrogen bonding between hydroxyl groups of ascorbic acid and histidine amino acid residues takes place and subsequently inhibits serine amino acid from participating in the charge relay system in enzymatic catalytic process (Graphical Abstract). The change of pH by ascorbic acid in the reactant was also considered for such an inhibitory effect. We tested the pH values of the original PBS and with the addition of ascorbic acid at different concentrations and found that the pH of the buffer solution reduced from 7.0 to 6.5 (2.5 mg/mL) and then 7.0 (1.25 mg/mL) of L-ascorbic acid. Although the optimal pH for trypsin hydrolysis was in the range of 8.0–8.5 [20], however in our experiment despite lower pH (around 7.0) we noticed the desired level of hydrolysis of HSA in enzyme control groups (Fig 6).

In this study, in both indicator systems, different doses of ascorbic acid were used which was ranging from 2 mg (113.6 mM), 1 mg (56.8 mM), 0.5 mg (28.4 mM), 0.25 mg (14.2 mM), and 0.125 mg (7.1mM) per reaction. The 50% inhibitory effect of L-ascorbic acid on cell monolayer was obtained at 0.125mg/reaction (1.25mg/mL) in the presence of 0.25mg ET solution while >85% inhibition was achieved at an equal concentration (2.5mg/mL) of L-ascorbic acid and ET (2.5mg/mL) in solution. A similar inhibitory effect of L-ascorbic acid was also observed in the second indicator system where human serum albumin is used as protein substrate and the highest level of interactions between L-ascorbic acid and ET was found best at 40°C compared to 37°C, although in both groups, the maximum hydrolysis and/or inhibition of HSA was achieved after 10 minutes of treatment. This finding is supported by the previous report [20].

L-ascorbic acid has been shown to have antiviral activity for more than half a century, including the work of the two-time Nobel Laureate Linus Pauling (Pauling, 1970). Vitamin C is a six-carbon and potent electron donor (2 and 3 hydroxyl groups) compound [25, 26]. It is available both in synthetic form and natural forms (citrus fruits and green leafy vegetables). The revival of interest in Vitamin C therapy for acute inflammatory disorders, grounded in sound biological rationale, follows decades of research. Emerging literature suggests that L-ascorbic acid may also play an adjunctive role in the treatment of a variety of viral infections [27]. Several observers including Linus Pauling have suggested in the past that L-ascorbic acid in high dosages is directly virucidal [27–31]. The inhibitory effects of ascorbic acid due to its strong binding and/or inhibition of proteases in viral infections are well reported, however exact mechanism of action has not been clearly stated [32]. Moreover, the role of trypsin in pancreatic cancer is also reported and our current findings of trypsin inhibitory effect by ascorbic acid could be an important area to work with such important area [33–35]. On top of our internal serine proteases, the external sources in the form of allergens from plants [36–38], pathogens [39–44], fungus [45, 46], protozoa [47, 48], arthropods [49], and poisons from reptiles [50, 51] are potential health hazards for human being and could be handled if proper doses of ascorbic acid are taken on time.

Through our findings, we have proposed an understanding regarding ascorbic acid’s role as SERPINs in addition to its role in regulating of immune system. In complement and blood coagulation cascades, serine proteases are actively involved and their overactivation could lead to the formation of disseminated intravascular coagulation [52]. Ascorbic acid plays an important role in handling such situations [53, 54]. Such a role may be attributed to the SERPIN activity of ascorbic acid.

One of the limitations of our study is that we have not been able to test other serine proteases. Moreover, it can be observed that L-ascorbic acid does not inhibit all serine proteases identically. As was observed in our study, though there was a 100% inhibition of ET, in contrast, there was only 58.50% inhibition for elastase. We have not been able to identify the reason, though we think it may be due to the differences in the purity of the ET and elastase. Furthermore, validation of our observations with L-ascorbic acid also needs to be compared and validated with other commercially available SERPIN.

## Supporting information

S1 Fig (TIF)

S2 Fig (TIF)

S3 Fig (TIF)

S1 Raw images (PDF)
