# Supplementary figures and images for: Ascorbic acid as serine protease inhibitor in lung cancer cell line and human serum albumin
Source: PLoS One. 2024 Jul 23;19(7):e0303706. doi: 10.1371/journal.pone.0303706 (PMC11265676; doi:10.1371/journal.pone.0303706)

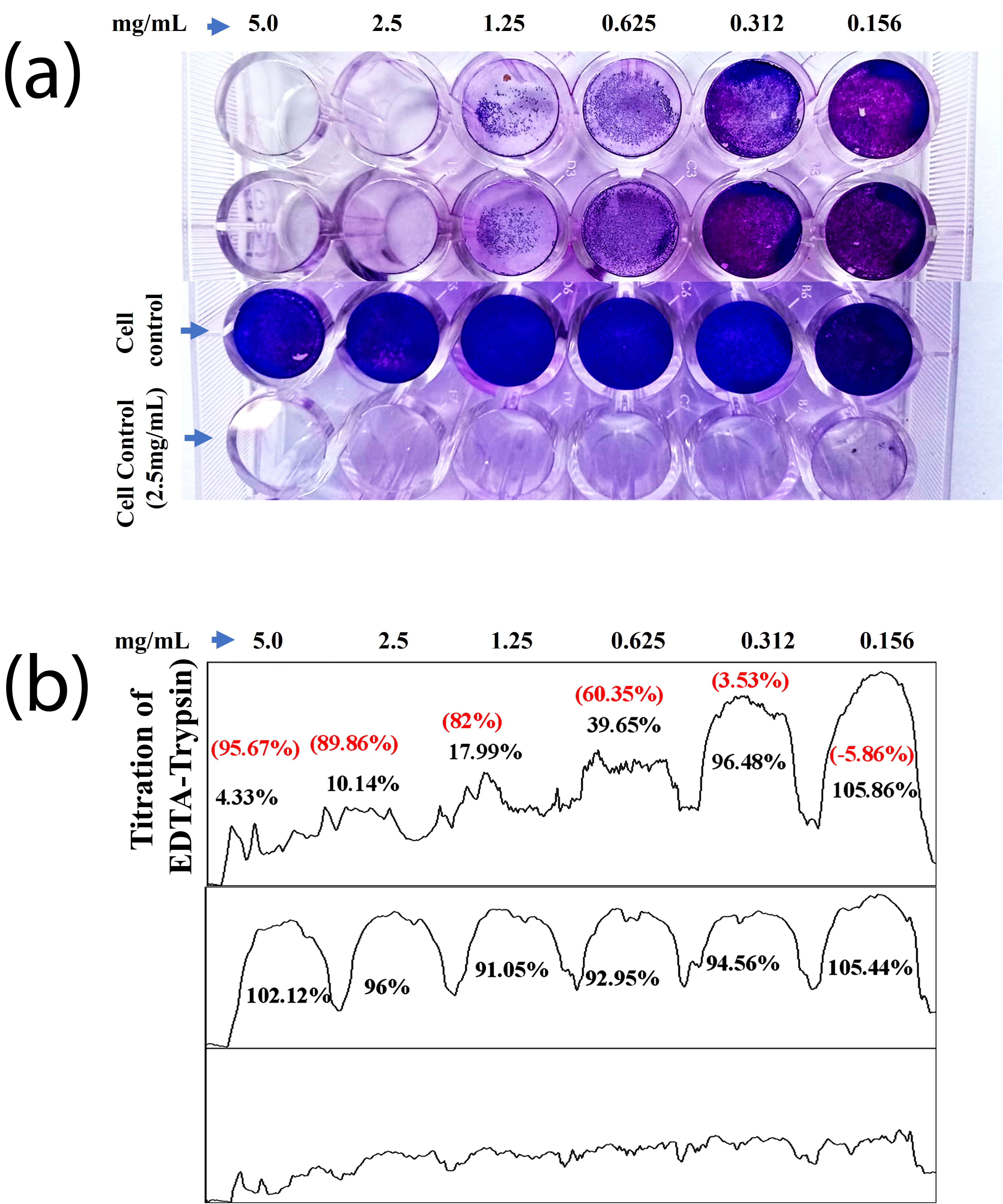

Supplement: S1 Fig — (TIF) [file pone.0303706.s001.tif]

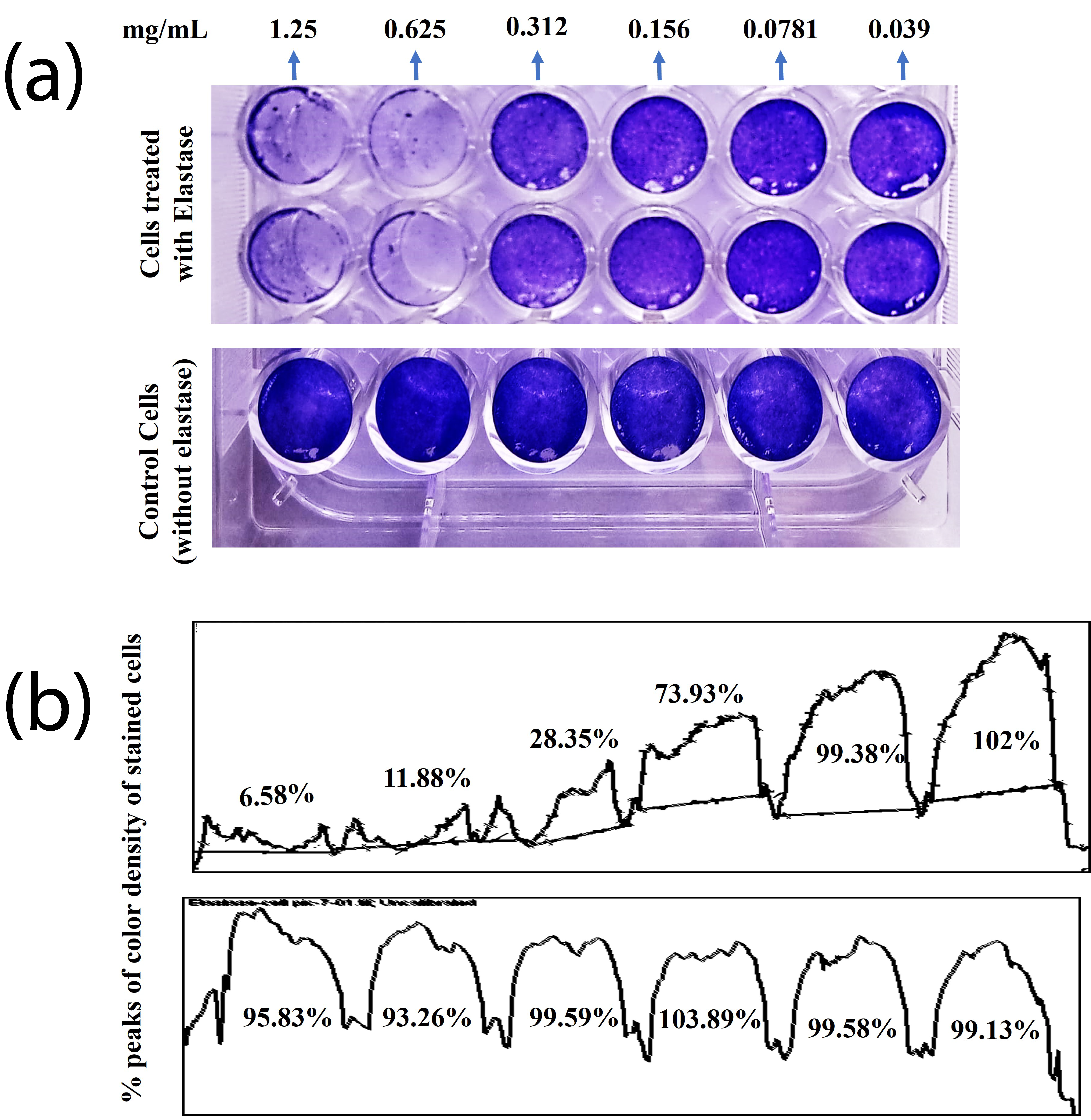

Supplement: S2 Fig — (TIF) [file pone.0303706.s002.tif]
