# Supplementary material for: Ascorbic acid as serine protease inhibitor in lung cancer cell line and human serum albumin
Source: PLoS One. 2024 Jul 23;19(7):e0303706. doi: 10.1371/journal.pone.0303706 (PMC11265676; doi:10.1371/journal.pone.0303706)
Supplement: S1 Raw images — (PDF) [file pone.0303706.s004.pdf]

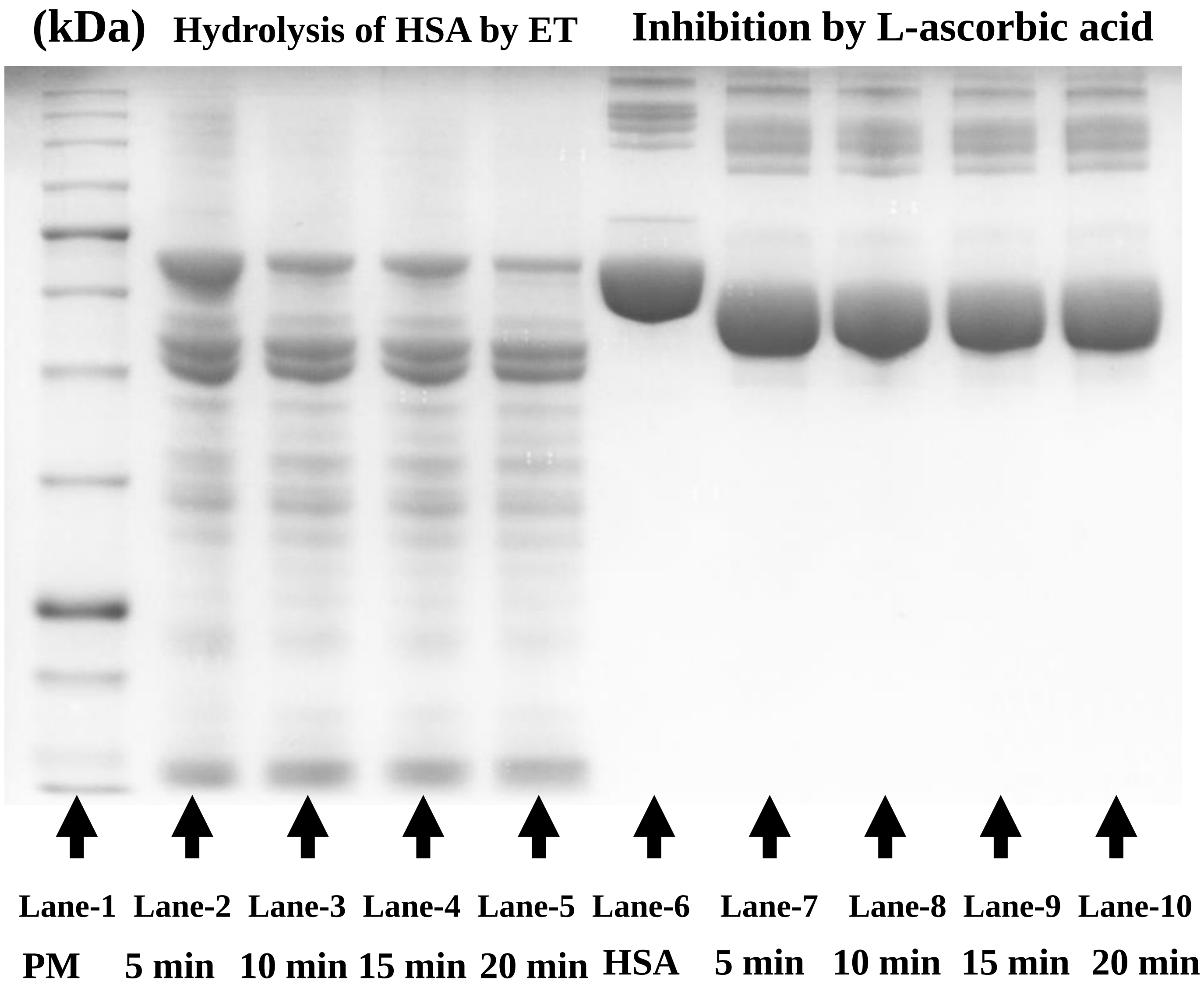

Fig. S1 Unedited Gel: The above slide is the unedited SDS-gel for the generation of Figure 5a (Lane-1, Lane-2 to Lane-5) and Figure 8a (Lane-1, Lane-6 to Lane-10). The HSA was exposed at temperature **37°C**.

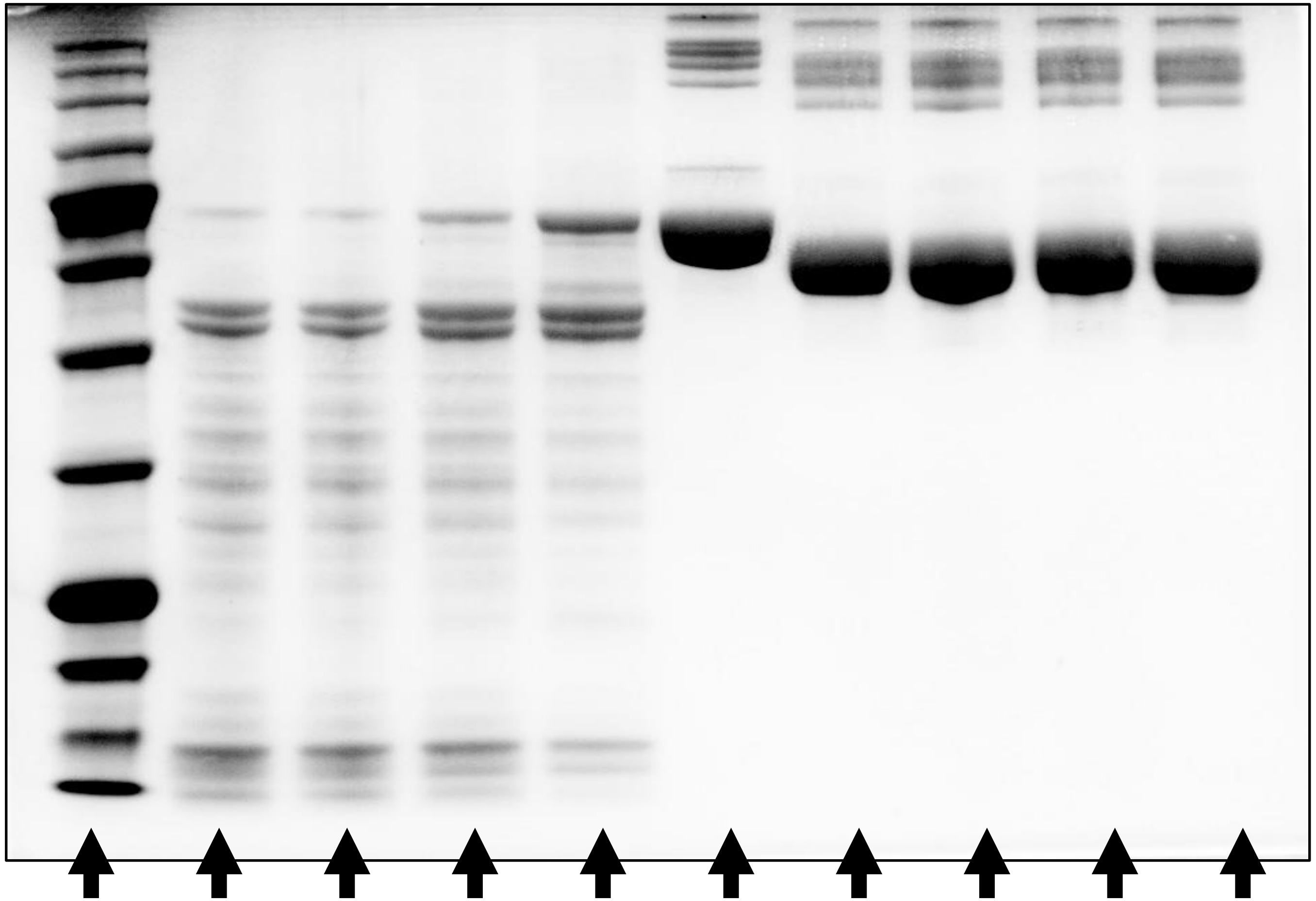

Fig. S2 Unedited Gel : The above slide is the unedited SDS-gel analysis for the generation of Figure 5b (Lane-6, Lane-2 to Lane-5) and Figure 8a (Lane-6, Lane-7 to Lane-10). The HSA was exposed at temperature **40°C.**

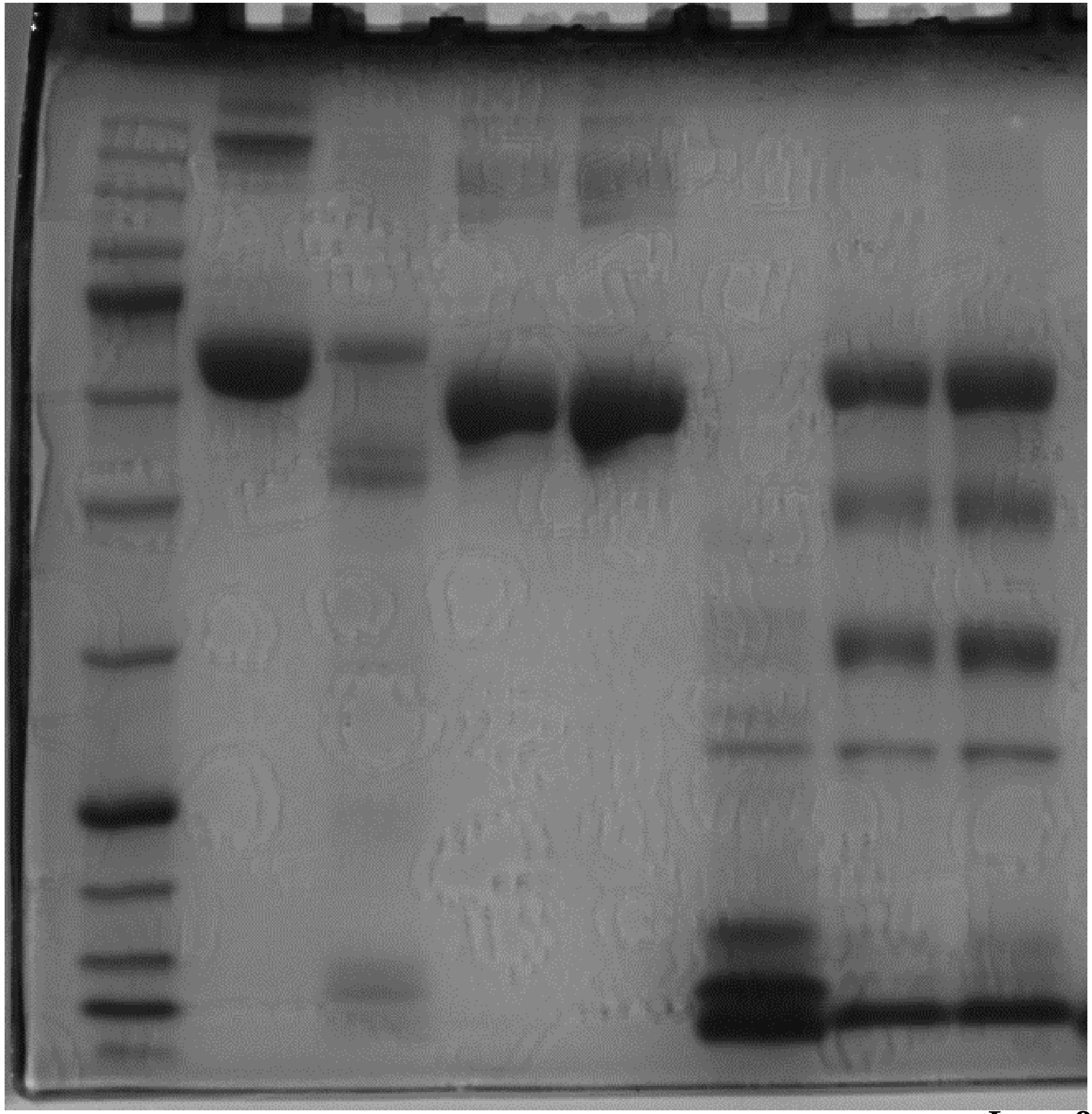

Lane-1	Lane-2	Lane-3	Lane-4	Lane-5	Lane-6	Lane-7	Lane-8
PM	HSA	HSA+ET	HSA+ET +AA	HSA+ET +AA	HSA+EL	HSA+EL +AA	HSA+EL +AA

Fig. S3 Unedited Gel: The above slide is the unedited SDS-gel analysis for the generation of Figure 7a (Lane-1, Lane-2, Lane-6, Lane-7 and Lane-8). Lane-3 was HSA treated with trypsin while Lane-4, and Lane-5 was HSA treated with EDTA-trypsin and ascorbic acid for comparison purpose. (comparative gel analysis by trypsin and elastase)

ET=EDTA-Trypsin
EL= Elastase
AA= L-ascorbic acid
